# Trauma-informed care approach in developing companion robots: a preliminary observational study

**DOI:** 10.3389/frobt.2025.1476063

**Published:** 2025-03-31

**Authors:** Keren Mazuz, Ryuji Yamazaki

**Affiliations:** ^1^ The School of Management, Hadassah Academic College, Jerusalem, Israel; ^2^ Faculty of Glocal Policy Management and Communication, Yamanashi Prefectural University, Kofu, Japan

**Keywords:** human-robot interaction, trauma-informed care, companion robot, humanoid robot, observation study, PTSD, elderly trauma survivors

## Abstract

**Introduction:**

This study explores the integration of Trauma-Informed Care (TIC) principles into the development of companion robots for elderly trauma survivors, particularly those with Post-Traumatic Stress Disorder (PTSD). The psychological effects of trauma, especially in aging populations, can complicate mental and physical health, highlighting the need for tailored technological solutions.

**Methods:**

The research involved two focus groups with Holocaust survivors (N = 12) who engaged directly with a social robot. Discussions explored their needs, barriers, and coping strategies based on their longitudinal experiences of trauma, resilience, and aging. Transcripts were thematically analyzed, identifying key TIC-related themes—safety, trust, self-compassion, and self-efficacy—in relation to engagement with companion robots.

**Results:**

Findings revealed that trauma survivors face significant challenges in communication and technology use. Safe and trusting environments were fundamental for meaningful engagement with robots. Self-compassion and self-efficacy emerged as essential to overcoming initial barriers, indicating that TIC-informed design features could facilitate better uptake and acceptance.

**Discussion:**

The study emphasizes the importance of incorporating TIC principles to ensure these robots meet the complex needs of trauma survivors. Findings also underscore the personal histories, ongoing changes in recollecting the trauma, and the need for stable, empathetic interactions highlights the complexity of designing assistive robots for profoundly affected populations. This study contributes to digital mental health and aging technologies by emphasizing stability, empathy, and user-centered design in developing assistive robotics as a universal, scalable solution.

## 1 Introduction

The psychological effects of traumatic events are extensive and may manifest themselves in advanced age. In the elderly, Post-Traumatic Stress Disorder (PTSD) can exacerbate the challenges associated with aging, which can further complicate trauma survivors’ mental, emotional and physical health (Qi et al., 2016).

In older adults, PTSD presents unique challenges. The U.S. National Center for PTSD reports that 70%–90% of individuals aged 65 and older have experienced at least one traumatic event, yet the prevalence of current PTSD in this age group is relatively low, ranging from 1.5% to 4%. However, 7%–15% exhibit subclinical levels of PTSD symptoms do not meet the full criteria for diagnosis.^1^

Lapp et al. (2011) highlight that PTSD can manifest long after a traumatic event, even decades later, particularly in older adults. The progression of PTSD varies significantly among individuals; symptoms may fluctuate, remain constant, or seemingly vanish for years before resurfacing in later life. This variability is influenced by numerous factors and interacts with the natural aging process, potentially accelerating age-related changes. According to O'Malley et al. (2023) PTSD can increase the risk of accelerated aging, dementia, and all-cause mortality. This may complicate the medical care of older veterans and are linked to diabetes, liver and heart disease, high cholesterol, stomach ulcers, and chronic pain (Watson, 2019).

Recent studies identify Holocaust survivors and veterans as having endured significant traumas affecting their later years, yet they also point to additional sources of trauma for older adults, such as bereavement (Elklit and O’Connor, 2005), physical injuries from falls (Chung et al., 2009), and surviving severe illnesses like COVID-19 (Sarangi et al., 2021).

Additionally, secondary trauma can reactivate PTSD symptoms (Bramsen et al., 2006). Delayed onset in older adults may result from underreporting, delayed referral, exacerbation of subclinical PTSD, or misdiagnosis (Lapp et al., 2011). Trauma’s impact extends beyond individuals, affecting subsequent generations socially, psychologically (Sweeney et al., 2018), and potentially epigenetically (Yehuda et al., 2016; Yehuda et al., 2009). Trauma trajectories into old age vary, with some survivors experiencing symptom stability or recovery, while others require ongoing intervention (Goldstein et al., 2024; Kelly et al., 2023).

O'Malley et al. (2023) argued that as the population ages, more individuals will receive care in long-term facilities, increasing the risk of worsening PTSD if staff lack the necessary skills to address symptoms or reduce retraumatization. This understanding has driven the integration of Trauma-Informed Care (TIC) into healthcare service provision and the practice of healthcare staff (Sweeney et al., 2018).

TIC approaches have developed in response to research demonstrating that trauma is prevalent across society, and as it is associated with mental health, it is a costly public health issue (Sweeney et al., 2018). Elliot et al. (2005) emphasize that TIC principles should be adopted as best practices, treating all service users as potential trauma survivors, regardless of theirm diagnosis.

In contrast to trauma and PTSD-specific services, Sweeney et al. (Sweeney et al., 2018) argue that “trauma does not need valid and reliable diagnosis or measurement, because principles of engagement are implemented for all service users, regardless of whether they have survived trauma. Trauma-informed approaches are, in effect, a process of organizational change that creates recovery environments for staff, survivors, their friends and allies, with implications for relationships” (pg.323). If trauma-informed principles are not adhered to, it is likely that trauma survivors will be unable to engage with services (Elliot et al., 2005).

Given the intricate interaction between trauma, PTSD, and aging, it is crucial to incorporate TIC principles in the development of assistive technologies. Laban et al. (2022) highlighted the potential of social robots as valuable tools for diagnosing and treating PTSD in older adults.

This is particularly important due to ongoing research on robotics-based interventions and robot-assisted therapy (Goda et al., 2023; Robinson et al., 2020; Robinson and Kavanagh, 2021a; Márquez-Sánchez et al., 2021). Robot-assisted therapy, a non-pharmacological approach, involves the use of robots to improve the health and psychological wellbeing of individuals through companionship. It has proven effective in sensory stimulation and managing emotional, behavioral, and psychological symptoms of dementia (BPSD), enhancing the quality of life for elderly patients (Márquez-Sánchez et al., 2021). As the use of Robot-assisted therapy grows in treating BPSD and mental health disorders, it becomes crucial to better understand the unique needs of aging trauma survivors and assess the effectiveness of these robotics-based interventions through co-design processes (Mazuz and Biswas, 2022; Östlund et al., 2020).

However, while existing literature has shown the promise of robot-assisted therapy for older populations in alleviating loneliness, support daily activities, and offer cognitive and emotional stimulation (Sawik et al., 2023), there remains a limited focus on how to integrate TIC principles directly into robotic design. Our approach diverges by emphasizing user-centered co-design methods specifically tailored to the complex emotional and psychological aspects of older trauma survivors. While user acceptance and general psychological wellbeing are studied, it does not delve into nuanced mental health needs related to trauma or PTSD among older adults. This study underscores the urgent need for TIC-aligned technologies that proactively address barriers of trauma survivors face as they age, such as trust, re-traumatization triggers, and communication challenges. This approach not only has the potential to create more supportive companion robots but also lays the groundwork for developing robot-assisted therapy as a non-pharmacological intervention.

In this paper, we aim to investigate the needs and barriers of trauma survivors face as they age and inquire how a social robot, a novel and promising technological solution, can assist in overcoming these barriers or suggest improved services for the companion of trauma survivors. We conducted in-person two focus groups with older adult Holocaust survivors who discussed their longitudinal perspective and experience of trauma, resilience and aging while engaging directly (rather than via video) with a social robot. As opposed to studies using videos of robots, the presence of the robot in this study and operation of the robot by the participants facilitates a lived experience and engagement and enables them to imagine themselves as regular users of the robot and to understand its value and limitations.

This article discusses these findings in relation to TIC approaches and principles. The findings will contribute to the growing body of literature on the use of robotics in trauma, mental health and elderly care, offering evidence-based recommendations for future development of robotics companionship.

## 2 Materials and methods

### 2.1 Methods

This study involved 12 elderly Holocaust survivors participating in two focus groups at social clubs in Israel. As part of the discussion in the focus groups, the participants were asked to interact with a social humanoid robot called RoBoHoN (developed by Sharp Corporation) in its commercially available, ready-made version without any technological modifications suitable for trauma survivors.

The sole criterion for inclusion in this study was their recognition as Holocaust survivors, acknowledging that such recognition entails exposure to significant trauma and eligibility for special rights and services due to their PTSD.

All of the participants were officially recognized as Holocaust survivors under Israel’s Nazi Persecutions Disabled Persons Law of 1957. This legislation provides compensation and support to individuals who suffered trauma and disabilities due to Nazi persecution during World War II. It acknowledges the trauma endured by survivors and offers financial assistance and benefits, including monthly allowances, medical services tailored to their specific needs, and additional support such as housing assistance and tax exemptions.

To gain recognition under this law, individuals must submit an application with documentation evidencing their persecution and resulting trauma and disabilities. The process involves medical evaluations and historical assessments to verify claims. Beyond Israeli state support, Holocaust survivors may also receive compensation from Germany, primarily through agreements facilitated by the Conference on Jewish Material Claims Against Germany (Claims Conference).

According to Ayalon (2005), research indicates that approximately 46.8% of Holocaust survivors exhibit signs of PTSD several decades after the Holocaust. Additionally, survivors report higher levels of depression, anxiety, and physiological symptoms compared to the general population. The long-term health impacts of the Holocaust are extensively documented, with many survivors suffering from PTSD symptoms. Furthermore, research has shown that the negative effects of the Holocaust extend beyond the survivors’ generation, affecting the second and third generations as well.

Morgan et al. (2022) have examined the long-term effects of the trauma on survivors, highlighting persistent trauma triggers, nightmares, hypervigilance, survivor’s guilt, and grief. These challenges are intensified by the typical difficulties associated with aging (pg.112). Despite these adversities, Holocaust survivors have been found to have significant resilience.

Thus, Holocaust survivors, indeed some of the remaining few who are living, offer a distinctive and invaluable longitudinal perspective on trauma, aging and resilience and could contribute significantly to the development of assistive technologies. Their perspectives can help developers understand the nuances of trauma responses, such as triggers and coping mechanisms, ensuring that robots are equipped to handle interactions with sensitivity and care.

#### 2.1.1 The research’s participants: Older adults trauma survivors

Participants were recruited through a local social clubs. Potential participants were provided information about the study through the club’s manager and encouraged to register to a focus group for an opportunity to engage with a robot and to share their perspectives.

As described in Table 1. All 12 participants (10 female; 2 male) were oldest-old Holocaust survivors between the ages of 82 and 92-years-old, with an average age of 86. All participants had children; 83% were widowed, and 75% lived alone. Despite their traumatic pasts, they rebuilt their lives, raised families, and pursued various professions. The participants are community dwellings, living independently in their own homes and maintaining independent functional and cognitive status. They regularly attend the social clubs.

**TABLE 1:** Summary of the participants socio-demographic characteristics.

	Age (In 2024)	Year of Birth	Country of Birth	Gender	Marital status	Do you have children?
Group A						
1	90	1934	Czechoslovakia	F	Widow	Yes
2	86	1938	Romania	F	Widow	Yes
3	83	1941	Romania	F	Widow	Yes
4	82	1942	Czechoslovakia	F	Married	Yes
5	92	1932	Ukraine	F	Widow	Yes
6	88	1936	Romania	M	Widow	Yes
Group B						
7	80	1944	Hungary	M	Married	Yes
8	90	1934	Iraq	F	Widow	Yes
9	86	1938	Iraq	F	Widow	Yes
10	94	1930	Czechoslovakia	F	Widow	Yes
11	87	1937	Russia	F	Widow	Yes
12	88	1936	Poland	F	Widow	Yes

All the participants came from working-class families, and, in the Nazi Holocaust, all of the survivors lost their parents, and half lost siblings. They immigrated to Israel from 1949 onwards. All of the participants had different levels of exposure to traumatic experiences between 1930-1945 including, hiding and imprisonment in concentration and death camps as subjects to Nazi persecution.

Notably, participants were not required to provide diagnostic documentation of PTSD or other mental health conditions, nor were they asked to complete diagnostic questionnaires. The sole inclusion criterion for this study was the participants’ recognition as Holocaust survivors, acknowledging that such recognition entails exposure to significant trauma and eligibility for special rights and services.

Despite the passage of many years, all participants acknowledged that the memory of their trauma continues to accompany them. Most of them reported persistent trauma and PTSD triggers of recurring dreams and nightmares. Additionally, they shared that they are still struggling with how and what aspects of their survival stories to share with their grandchildren.

#### 2.1.2 The research’s site: the social club

The study was conducted in two social clubs in Israel, which served as community-based research sites. Social clubs for the elderly in Israel are formal social services designed to enhance the quality of life for older adults. These clubs are operated by local municipalities 5 days a week from 8 a.m. to 1 p.m. They primarily serve as a social framework to alleviate loneliness and provide recreational activities. Additionally, they are valuable resources for promoting active aging through self-empowerment programs and community volunteering opportunities.

Physical exercise is the most common activity at these clubs, followed by lectures on various topics, art classes, and table games. Light refreshments and breakfast are provided. Members pay a monthly fee averaging NIS 60 (17USD), with additional fees for some activities based on participation.

The staff in this particular clubs includes a manager, a woman in her 60s with over 20 years of experience in elderly care, and a caregiver, a woman in her 50s with over 15 years of experience. These caregivers provide companionship and personal and social care.

#### 2.1.3 The research’s social humanoid robot: RoBoHoN

This study employed Sharp Corporation’s RoBoHoN SR-05M-Y, a commercially available humanoid robot with integrated smartphone capabilities, without any technological modifications.^2^

RoBoHoN is a compact, robot-shaped smartphone standing approximately 19.5 cm tall, equipped with standard mobile phone functions. It operates on the Android 5.0 operating system, which can be navigated using a 2-inch touchscreen located on its back. The device features built-in speech-to-text (STT) and text-to-speech (TTS) servers that facilitate speech recognition and production, enabling voice as its primary interface. RoBoHoN’s communicative functions include responding to the owner’s voice, answering questions, and executing actions based on voice commands. The robot can perform human-like movements such as nodding, making expressive gestures, dancing in sync with conversations.

In previous academic studies, RoBoHoN has been utilized to explore its applications in healthcare and human-robot interaction with older adults. For instance, Chen et al. (2021) investigated its role in assisting elderly individuals with mild cognitive impairment, while Fiueroa et al. (2023) examined its effectiveness as a social robot for older adults with cognitive decline. Additionally, Yamazaki et al. (2023) conducted studies on its impact on older adults living alone.

In this study, during focus group sessions, the robot was introduced to participants and demonstrated its abilities to move its arms and head autonomously, singing, dancing, and conducting exercises with the participants. As part of the robot’s demonstration, the participants asked it to sing and dance and the robot replied, “I get it, I will dance/sing (name of the song/dance)”. At the end, the robot added “dancing is so much fun”.

Its functionalities are powered by voice recognition software and a speaker, while LED lights in its eyes and mouth provide visual indications of animation and speech. Movement is achieved through the coordinated actions of its arms and head. The robots speak Japanese and English.

### 2.2 Observational study methods

#### 2.2.1 Data collection and procedure

This qualitative research employed a user-centric participatory design approach, incorporating two focus groups with observations and in-depth, open-ended interviews conducted by the principal investigator. As described in Table 1 each focus group comprised six participants who were already acquainted with each other (each focus group extended for 1 h and half). Due to its focus on depth and the lived experiences of the participants, an appropriate sample size ranges from three to six individuals. This approach prioritizes a detailed exploration of the participants’ subjective narratives. Therefore, the focus group method is adequate for exploring and assessing the potential long-term impact of a social service robot (Sawik et al., 2023).

The sessions were held in one of the rooms of the social club, with the robot positioned centrally so that all participants were able to see and interact with it. Chairs were arranged in a circle around the robot which was connected to an electrical socket and wireless internet. The manager of the club introduced the research and the robot to the participants and explained the research. Participants signed informed consent forms to participate.

At the beginning of the session, each participant provided a brief self-introduction, including their age, education, occupation, family status, country of birth, and their experiences during the Holocaust. To foster open dialogue and encourage free associations, the researcher refrained from asking direct questions. Instead, the researcher demonstrated the robot’s capabilities by instructing it in English to sing and dance.

Participants observed the robot, touched it, and attempted to instruct it themselves to sing and dance, and observed how the others interacted with the robot. These interactions prompted more conversations among participants about various aspects and goals of technology, such as their current barriers to using smartphones, questions about whether the robot would replace smartphones, and concerns about the cost of technology.

After 10–15 min of robot activity, the researcher facilitated a discussion using a semi-structured interview format with nine open-ended questions referring to three categories such as needs, barriers and solutions (strategies to overcome barriers) and based on the robot’s demonstration:

⁃ Needs:
  • What are their current social and health needs related to their aging and trauma?
  • What enables them to be open and talkative with others about trauma triggers and memories and to receive aid and companionship?
  • What types of technology do they need and use daily for health purposes?
⁃ Barriers:
  • What barriers prevent them from receiving treatment, compassion, and care as they age?
  • What challenges do they currently face in using technology?
  • How could RoBoHoN help them overcome these barriers?
⁃ Solutions:
  • How do they address and overcome problems and barriers in receiving care?
  • How might RoBoHoN assist them at home with trauma triggers?
  • What crucial functionalities of RoBoHoN do they value or desire to help manage and cope with their trauma?

As participants responded, further questions and discussions ensued. This method allows for the exploration of interactions between participants and the robot. The presence of the robot and operation of the robot by the participants facilitates a lived experience and engagement and enables them to imagine themselves as regular users of the robot and to understand its value and limitations. As previously discussed, experiences and reactions to traumatic events vary significantly among survivors. Focus groups serve as a valuable qualitative research method, facilitating discussions on both similarities and differences among participants.

During the sessions, audio recordings were made, and extensive field notes were taken immediately after each focus group. These were then transcribed. The researchers translated the notes into English and clustered the themes accordingly.

#### 2.2.2 Data analysis

The discussions underwent an iterative process of thematic analysis to identify common and shared topics that emerged from the data. These topics reflected shared experiences among all participants, coded into three categories: Needs, Barriers and Solutions (strategies to overcome barriers).

Then, axial coding was conducted to condense the subthemes, which resulted in second-order constructs of four themes: safety, trust, self-compassion, and self-efficacy.

These themes emerged across the different categories, whether the participants discussed their presence or absence. For example, trust was significant as a need and as a barrier (i.e., lack of trust hindering service reception). After mapping the connections between the themes, they were categorized together based on their dynamic and relational interplay with TIC principles, as summarized in the following Table 2.

**TABLE 2:** Comparison between the themes of this research and Trauma-Informed Care (TIC) principles and the suggestions for development of a companion robot.

Themes and Subthemes	TIC principles (Sweeney et al., 2018; Levenson, 2020)	Suggestions for a companion robot development
Safety and Trust • The need for a physical and emotional safe environment to share traumatic experiences • Trust as a foundation for recovery, storytelling, and interaction • Trust as both a need and a barrier when absent • Fear of judgment and shame preventing survivors from speaking • The importance of empathetic, non-judgmental, and respectful communication • Control over storytelling as a key factor in feeling safe • Transparency and Empowerment- accepting the changing nature of trauma stories over time and that there are new recollections and reinterpretations of experiences with age • The dynamic and fluctuating experience of trauma, even decades later • Lifelong influence of trauma, regardless of time passed • The significance of validation and emotional support over the years • Uninterrupted open communication facilitated but not controlled by the robot	• Seeing through a trauma lens • Safe relationship and safe environment • Trustworthiness and transparency • Sensitive discussions about trauma • Appreciation of invisible trauma and intersectionality • Pathways to trauma-specific support	1. Facilitating Safe and Trusting Interactions • Robots can become mediators for safe and trusting interactions • Create a safe environment by fostering non-judgmental, empathetic communication • The potential of robots to facilitate sharing is by being non-judgmental • Use open-ended questions rather than interrogative approaches to validate feelings and encourage dialogue • Recognize the feelings of loss, shame, and fear that often accompany trauma survivors • Appreciate invisible trauma and the intersectionality of individual experiences • Allow users to share at their own pace, providing autonomy and respecting boundaries in storytelling • Avoid confrontation and coach self-regulation to support emotional stability • Always ask and use person-first language to prioritize dignity and empathy 2. Providing Meaningful Companionship • Robots as companions able to provide daily interaction and memory recording • Balance companionship with privacy and consent, ensuring that memories are recorded only with explicit permission • Provide access to trauma-specific services upon request, linking users to appropriate resources • Enable robots to engage in meaningful interactions that support users’ emotional needs while respecting their privacy 3. Ensuring Trustworthiness and Pathways to Support • Build trustworthiness through reliable and stable systems to ensure consistent interaction • Always provide support if the robot malfunctions or loses connectivity • Address concerns about reliance on infrastructure, ensuring functionality even without internet access • Be transparent about the robot’s capabilities and limitations to manage user expectations effectively • Offer pathways to trauma-specific services to ensure users receive appropriate support when needed
Self-compassion and Self-efficacy • Forgive oneself for surviving trauma • Live productively • Using proactive behaviors to survive challenging circumstances • The importance of being valued for independence and abilities • The need for appreciation beyond surviving trauma • The ability to adapt to new technologies as a key to resilience • Using technology to find purpose and remain engaged in society • Resilience building • Positive thinking about their capabilities • Imagination, and empathy • To encourage agency and self-efficacy • Encourage socialization - social engagement and social interaction (play)	• Empowerment, choice, and control • Collaboration and mutuality • Survivor partnerships	1. Empowerment, Choice, and Control • Use strengths-based approaches to empower individuals and support them in taking control of their lives despite the trauma • Validate that it can be hard and scary to find oneself in a position of needing help • Emphasize resilience and adaptations to trauma rather than focusing solely on symptoms (this is the oscillation) • Encourage users to use the robot as a tool for memory recording, self-expression, and self-discovery while maintaining control over privacy and consent • Recognize the importance of a functional and embodied appearance for robots to meet user expectations and enhance their perception of usefulness 2. Collaboration and Mutuality • Foster mutual and collaborative relationships by ensuring the robot facilitates non-judgmental, empathetic communication • Use robots as mediators for reflection and imagination, helping users process trauma in creative and engaging ways • Enhance social interactions by encouraging meaningful connections with others, including younger generations, through playful and engaging features • Support shared experiences and evoke positive memories to deepen mutual understanding and collaboration 3. Survivor Partnerships • Enable the robot to engage in activities that support resilience, such as storytelling, assisting with daily tasks, and providing companionship • Develop features that enhance the user’s ability to adapt and interact, such as creative play, games, and tools for self-expression • Design robots to act as companions of therapeutic tools like toys, fostering creativity and playfulness in processing trauma • Collaborate with trauma survivors in the design process to ensure the robot meets their needs and aligns with their preferences

## 3 Results

The following sections elaborate on the intersection between the four themes.

### 3.1 Safety and trust themes

In discussing and recounting their traumatic experiences, the survivors emphasized the immediate need to feel safe and be able to trust others. Building safe environments and trusting relationships is crucial not only for recovery and treatment but also for the process and interaction of storytelling as witnesses.

One of the nuanced mental health needs that emerged was the emphasis on safety. All participants agreed that they could only reveal their stories when they felt safe. Otherwise, many did not speak about their experiences for years. Safety is a need and when it is absent it is a barrier to receive aid and compassion from others. As one woman said, “If I do not feel safe and supported in discussing the event, it can lead us to feeling guilt or shame because we survived.” Another woman added, “At the beginning, we felt that others judged us and that our story was too much to bear, so we did not speak—not to our families and not to other survivors. As, if you do not speak, you can overcome it.”

They agreed that safety involves non-judgmental, empathetic, respectful, and supportive communication, allowing them to talk at their own pace, refuse to answer questions, or stop the conversation when they want to–allowing participants some control over how they share their experiences. Participants suggested that a robot should ask more open questions rather than specific and narrow ones, avoiding an interrogative approach and allowing them just to open up and talk freely. They noted that a nonhuman robot might be less judgmental than a human–facilitating the sharing of very personal and private experiences (not just with the robot but also as a mediator with others).

Another factor that fostered a safe and non-judgmental relationship was the understanding that their stories changed over the years as they recollected evidence or reinterpreted insights with age. One woman noted, “Today, years later, as we all aged, I find myself more talkative and able to speak of things I could not say before. But then others, even my children, ask me why I did not speak of these things before. I just could not back then.” This highlights that trauma is a living experience that fluctuates over time, regardless of how much time has passed since the event.

The adverse effects of trauma may have an immediate or delayed onset and may be in the short-term or lifelong. Experiencing and meaning-making around trauma are connected to an individual’s cultural beliefs, social support, trust, gender, age, and other factors. Thus, the need for safe and trusting interactions remains significant, even years after the traumatic event. Participants require validation of their experiences and emotional support to acknowledge their trauma and its impact over years (i.e., “that it has not gone over the years, it is still present”). Even if they have shared their story many times and written about it, they may now remember new details or interpret experiences differently.

Regarding the social robot, participants were interested in the companionship it affords because they were able to talk to it during the day, and it was able to record their memories. However, they emphasized the importance of privacy, and that robots should not share their stories without consent.

Another concern was the reliability of the robot. Participants were skeptical about its usefulness and dependability, as one participant noted, “If there is no Wi-Fi or internet connection, the robot dies, and then what?” This emphasizes the need for stable and continuous interactions to build trusted and reliable relationships. Dependability, especially on others or on infrastructures, which was lacking during the traumatic event, is crucial to feeling safe and trusting the robot.

### 3.2 Self-compassion and self-efficacy themes

Developing self-compassion and a sense of self-efficacy helps trauma survivors cope with their experiences and overcome the effects of trauma. Resilience is part of the coping strategy with trauma.

As one woman described, “When we do things successfully, it contributes to our self-image as productive and proactive people - that we can do things as normal.” The other participants agreed, emphasizing that their secret to long life after experiencing trauma is their self-efficacy and positive thinking about their capabilities. Almost all of the participants studied, built careers, and raised families with pride. They agreed that this was not possible without positive thinking and self-compassion - the ability to forgive oneself for being the sole survivor while others perished.

One man added, “During the war, it was not only one event to deal with but years of events during which we changed places and moved around. We had to be proactive to survive.” In relation to how others react to them, he continued, “Sometimes people think they need to help us and do not recognize our abilities.” This underscores their need to be appreciated and valued not just for surviving trauma but also for their ability to survive, their sense of independence, and self-efficacy. Against all odds, they have used their personal capabilities to direct and manage their own lives.

Capability and function were also important in perceptions about the robot. Almost all of the participants, upon seeing the robot for the first time, immediately asked, “What can it do?” They expected it to be useful and to perform valuable tasks that benefit the users. They kept asking for the robot to perform practical “real” tasks that every elderly person needs help with, such as “Does it cook? Does it clean? Does it go to the market?” One man asked with a laugh, “Can it produce children?” adding that this is the most important value for humankind.

Many were skeptical about what robots can actually do and were not impressed with the robot’s small size and the technology currently being tested. One participant explained, “When you see the robot with hands and legs, you expect it to walk and do things with its hands. Otherwise, it is a robot with little capability; it is of little value, little use. How can it be useful for my life as an older adult?” Another participant continued this point, saying, “At the concentration camp, we were selected with only one criterion: can we work or not, can we be useful or not. Those who looked strong and able were sent to work; the others sent to the camps. We always tried to be bigger than we were, we doubled our age.”

The concept of embodiment of a useful (functional) appearance was important to determine the value, worth and utility of the robot. The first impression of the robot’s physical appearance and size determined whether it might work or be useful. Others explained this point by referring to their post-war life: working and finding purpose, and always doing something beneficial and in service of others and society. They discussed, for example, how the TV “captured” people, making them watch passively for hours and waste time without interaction or the facility to make any contribution. Participants emphasized the need for more engaging and interactive robotic function–assistance with exercises, helping them connect with their grandchildren, or reading stories in their mother tongue (not Hebrew, but the language of their childhood), because not many people are left who can speak with them in their native language.

Some participants argued that while robots should assist the elderly, they should not take away human capabilities. One participant said, “If it will remember and think instead of me, how will I improve? What else can I do? It can only assist me but cannot take everything from me.”

The idea of self-efficacy is significant in reaffirming their resilience. One woman explained, “We all witnessed all kinds of technology from the oil lamp to today’s technology. Some have changed, and some are not available anymore.” All the others agreed, underscoring that they had lived through technological transitions involving TVs, radios, the internet, social media, and more, and understood the opportunities created by technology and how humans must adapt to it. One participant emphasized that the ability to adjust and change is the key to survival and resilience.

All participants agreed that the ability of the robot to engage in social interaction should be further developed, as it emphasizes the users’ self-expression, agency, playfulness and creativity - similar to playing with toys. One participant shared how they found comfort in toys: “Back then, we did not have toys. It could be a towel that we drew eyes on or something that we found in new shelters. Sometimes it was more valuable than food. My mother always looked for toys, and even now I still play.” The toy or the robot may mediate between the real world and the playing self and open a space for reflection and imagination which is important for processing trauma.

Others agreed that the main issue is the lack of games or toys for the elderly as a means of self-expression and efficacy. They suggested the robot offer them a new opportunity to play with and imagine. “Playing makes us more approachable and accessible to the young generation. It evokes memories and is much better than yet another medical device that looks scary and is not engaging.” One participant added, “I am glad that there are delays when the robot’s responses - just like me. It takes time to answer, so maybe other people can learn to be more patient with those who are different and disabled.”

## 4 Discussion

The findings outlines three key categories: Needs, Barriers, and Solutions of the older adults trauma survivors participants. These categories overlap resulted in four themes: Safety and Trust, and Self-compassion and Self-efficacy.

The intersection of needs and barriers highlights the critical role of safety and trust, which influence the participants both through their presence and absence. This suggests the importance of creating a safe, supportive, and trusting environment to help trauma survivors to overcome barriers in receiving treatment, compassion, and care as they age. Such an environment enables them to experience self-compassion and self-efficacy in coping with their trauma. There is a dynamic interplay between the external world—a safe and trusting environment—and the internal world of trauma survivors, as they navigate their own abilities to cope with trauma triggers and memories as they age.

The themes that were found in the group discussions reflect the participants’ needs, barriers, and solutions based on their life experiences as trauma survivors. Their engagement in this research may assist the understanding of the ways in which technology is adapted and accepted by older adults who are trauma survivors and help to close the gap between what designers and engineers think elderly users need, and what elderly trauma survivors as users of the robot ask for.

Unlike much of the existing research on technology acceptance, these findings focus on the nuanced emotional and mental health demands of older trauma survivors, rather than simply usability or convenience. The insistence on safety and trust—to the extent that many survivors remained silent for years—underscores how feeling unsafe can prevent both emotional expression and effective use of technology. Whereas safety is a concern in most user studies, here it is amplified by the legacy of trauma and PTSD, where re-traumatization risks are high. This emphasis on personal histories, ongoing changes in recollection and memories, and the need for stable, empathetic interactions highlights the complexity of designing assistive robots for profoundly affected populations.

Based on TIC approach and principles, we found the themes presented above overlap and fit with the TIC approach. Table 2 illustrates the overlap between the identified themes and TIC principles and their implications for developing social robots.

TIC is a process of administrative change aiming to create environments and relationships that promote recovery and prevent retraumatization (Sweeney et al., 2018; Levenson, 2020). TIC can be distinguished from trauma-specific services or treatment. In developing social robots as a universal and shelf product, it is important to embrace common themes as trauma is widespread and may be experienced differently over years.

### 4.1 The oscillation between themes

Based on the discussions and the comparison to TIC, there are oscillation between the themes.

Safety and trust can be seen as complementary, particularly in the context of TIC principles in creating secure environments for trauma survivors. Safety refers to the protection from harm or danger, while trust involves the user’s confidence in the reliability and integrity of individuals or systems (i.e., that the robot will function correctly). Both concepts are interdependent: safety measures often lead to increased trust, as individuals feel secure in their environment (including the robotic and technological infrastructures).

Conversely, trust is crucial for safety because people are more likely to follow safety protocols if they trust the source of the guidelines and the systems. In technology, users are more likely to adopt and use devices or systems they trust to be safe and secure. However, while safety and trust enhance each other, for the trauma survivors they are not identical and can sometimes diverge in practice. Effective safety measures do not automatically result in trust, especially if the measures are perceived as judgmental or overly restrictive and controlled by the robot.

Self-compassion and self-efficacy also can be seen as complementary, though they are distinct constructs. Self-compassion involves treating oneself with kindness, empathy and understanding during times of failure or difficulty, while self-efficacy refers to the belief in one’s ability to achieve goals and succeed in specific situations despite the trauma.

There is a positive association between self-compassion and self-efficacy, especially for trauma survivors. Self-compassion helps individuals recover from traumatic events and maintain positive thinking, which can empower them and enhance their self-efficacy by fostering resilience and confidence in their abilities. Conversely, higher self-efficacy can lead to increased self-compassion, as individuals with a strong belief in their capabilities are more likely to treat themselves kindly when facing challenges. However, while they are related and can support each other, they are not identical and represent different aspects of psychological wellbeing.

Developing a social and companion robot based on these principles emphasizes the oscillation between safety and trust and between self-compassion and self-efficacy. This oscillation reflects the lifelong emotional experiences of trauma survivors, who alternate between past and present, memories and affects, pleasant and unpleasant feelings, and the “pleasure principle” and the “reality principle” (Axmacher and Heinemann, 2014). This lifelong oscillation means that one state often interrupts the other. During extreme negative experiences of trauma, positive feelings may become inaccessible. Thus, “oscillations between positive and negative feelings are crucial for healthy development and clinical treatment” (28, pg. 142).

Through processes of containment (Bion, 1963) supported by empathic relationships—whether with a person or a robot—negative emotions can be integrated with positive experiences. A TIC approach in developing companion robots must acknowledge and incorporate these oscillations into its design, scripts and interactions. This approach not only has the potential to create more supportive companion robots but also lays the groundwork for developing robot-assisted therapy as a non-pharmacological intervention.

## 5 Limitations and future research

Adopting a TIC approach in developing companion robots is crucial, as it aims to enhance the health, emotional and psychological wellbeing of individuals through non-pharmacological, assisted therapy (Robinson and Kavanagh, 2021b; Márquez-Sánchez et al., 2020). However, these recommendations require long-term evaluation to understand the sustained benefits and potential challenges of social robot interventions. Future research could expand to include a larger and more diverse sample of individuals who have experienced various forms of traumatic stress and PTSD, offering a broader and more nuanced understanding of the robot’s effectiveness across different trauma scenarios.

Additionally, this study does not include elderly individuals with cognitive impairments or dementia, a significant and growing segment of the elderly population. Testing the social robots with trauma survivors and dementia patients is important to determine their effectiveness in addressing the specific needs and challenges faced by this group.

A key concern for the further development of robotics research is incorporating users’ intrinsic knowledge throughout the entire technological design process. This study emphasizes the importance of recognizing older adult trauma survivors as experts in their own lives and daily practices as technology users. Thus it is important to involve elderly users in the co-design of these technologies to ensure they meet their specific needs. Future research should include not only end-users, but also multiple stakeholders involved as secondary users, such as caregivers, healthcare employees, relatives, and local management at municipal social centers.

Future research should aim to address these limitations by including anthropological study of the “adaptive learning” frames (Mazuz and Yamazaki, 2023), a detailed study of ritualized and playful practices that could yield important insights about what it takes for companion robots to become ‘accepted’ and ‘trusted’ by users.

Such comprehensive studies are crucial to fully understanding both the potential and limitations of incorporating TIC principles into social robots, particularly from a technological and optimization standpoint.

Building companion robots that integrate TIC principles and nuanced emotional response systems necessitates advanced machine learning algorithms and robust data analysis—challenging tasks that must often be refined in real time (Sutikno, 2024). These constraints complicate the robot’s ability to interpret and respond empathically, a core requirement for TIC-based care. Further, although AI-driven robots can gather data through multiple sensors, the current state-of-the-art frequently lacks the precision needed to address sensitive emotional states in trauma care, reducing the robot’s capacity for truly trauma-informed interactions. One short-term solution that could advance research in this domain involves operating the robot remotely via a Wizard of Oz setup, integrating it into caregiver training programs that focus on TIC principles.

Moreover, when striving for efficiency and optimization, technological and managerial limitations become more pronounced, especially when attempting to integrate off-the-shelf robotic products into trauma-informed care. Many commercially available robots are not designed with TIC principles in mind, making their adaptation for trauma survivors particularly challenging. For instance, Sawik et al. (2023) describe a multi-criteria optimization model designed to balance three core objectives in elderly care robotics: maximizing care efficiency, maximizing robot utilization, and minimizing caregiver stress. The authors tested their mathematical model on a sample dataset comprising 100 older adults and 100 possible assistants (robots or human caregivers). They adjusted the weighting of each objective to see how tasks would be allocated; for instance, placing full emphasis on efficiency led to assigning all tasks to human caregivers, while prioritizing robot utilization or caregiver stress reduction shifted tasks entirely to robots.

Incorporating TIC principles adds another layer of complexity, introducing parameters related to emotional safety, trigger avoidance, and user autonomy, which can alter task distributions. On one hand, embedding TIC alignment yields solutions that better safeguard mental wellbeing by favoring either specialized robots with empathy modules or human caregivers trained in TIC. On the other hand, this approach demands more detailed data collection, higher equipment costs for advanced robot capabilities, and potentially slower integration into real-world settings. Nevertheless, programming robots according to TIC can alleviate psychological distress, reduce triggers, and foster a greater sense of control for trauma survivors, ultimately offering a more holistic and user-centered model of care.

Thus value co-creation between researchers, healthcare providers, and technology developers is vital to successfully implementing companion robots for trauma survivors. A sustainable business model is also critical, ensuring that these robots are not only technologically feasible but also financially viable and accessible for widespread use. Further research could focus on developing frameworks that integrate TIC-oriented features into optimization models, enabling automated systems to dynamically balance emotional, practical, and cost considerations for more adaptive, person-centered assistance.

## 6 Conclusion

This article argues that trauma can have long-lasting psychological effects that may resurface or worsen in old age. This study explores the integration of TIC principles into the development of companion robots for elderly trauma survivors, particularly those with PTSD. By combining human-robot interaction with trauma care, this research provides crucial insights into developing companion robots as a non-pharmacological interventions that support elderly trauma survivors in a thoughtful, user-centered manner, contributing to the broader field of digital mental health and aging technologies. This study makes several key contributions:

1. Novelty of the Topic and Participant Perspective: It addresses trauma and PTSD in the context of aging, a crucial yet underexplored area, particularly in elderly trauma survivors. Holocaust survivors—some of the few remaining individuals with lived experience—offer a unique and invaluable longitudinal perspective on trauma, resilience, and aging.
2. Methodological and Theoretical Contributions: By conducting in-person focus groups, this study allowed elderly trauma survivors to engage directly with a social robot, rather than through video-based observations. This hands-on approach provided deeper insights into their perceptions, interactions, and emotional responses, offering a more nuanced understanding of how trauma survivors relate to robotic companions.
3. Practical implications: the study offer practical suggestions for a companion robot development based on the comparison between the themes of this research and TIC principles (via Table 2.). The study also underscores the potential and limitations of integrating TIC principles into social robots, particularly from a technological and optimization standpoint. Many commercially available robots are not designed to meet trauma survivors’ specific needs, making adaptation difficult. Overcoming these barriers requires value co-creation among researchers, healthcare providers, and technology developers, as well as the development of sustainable business models to ensure long-term feasibility and accessibility of TIC-based social robots.

By integrating psychological, technological, and managerial perspectives, this study provides a foundation for future research on trauma-informed robotics. Addressing these challenges through co-design, AI-driven adaptability, and optimization models will be essential for creating scalable, ethical, and user-centered robotic solutions for elderly trauma survivors.

## Data Availability

The datasets presented in this article are not readily available because of ethics requirements. Requests to access the datasets should be directed to KM, kerenmaz@gmail.com.

## Footnotes

1 Retrieved from (accessed 12 January 2025): U.S. Department of Veterans Affairs. (n.d.). Posttraumatic stress symptoms among older adults: A review. National Center for PTSD. https://www.ptsd.va.gov/professional/treat/specific/symptoms_older_adults.asp

2 The use of Robohon is because it is part of a larger research project which aims to explore the potential effects of social robotics on communication with and between older adults with MCI. The robot is based on the Android version 8.1 operating system, the robot’s is 19.5 cm in height, weighs approximately 360 g. For more information about Robohon see: https://robohon.com/
